# Supplementary material for: Structural basis for antibiotic transport and inhibition in PepT2
Source: Nat Commun. 2024 Oct 9;15:8755. doi: 10.1038/s41467-024-53096-6 (PMC11464717; doi:10.1038/s41467-024-53096-6)
Supplement: Supplementary file 1 — Supplementary Information [file 41467_2024_53096_MOESM1_ESM.pdf]

Supplementary Material

Structural basis for antibiotic transport and inhibition in PepT2.

Joanne L. Parker *et al.*

*Corresponding author. Email: simon.newstead@bioch.ox.ac.uk

Supplementary Table 1. Cryo-EM data collection, refinement, and validation statistics

	RnPepT2 cefadroxil bound state (EMDB-44599) (PDB 9BIR)	RnPepT2 amoxicillin bound state (EMDB-44600) (PDB 9BIS)	RnPepT2 cloxacillin (pose 1) bound state (EMDB-44601) (PDB 9BIT)	RnPepT2 cloxacillin (pose 2) bound state (EMDB-44602) (PDB 9BIU)
**Data collection and processing**				
Magnification	165,000	165,000	165,000	
Voltage (kV)	300	300	300	
Electron exposure (e–/Å^2)	54.8	55.3	57.6	
Defocus range (μm)	-2.5 to -0.8	-2.0 to -0.5	-2.0 to -0.5	
Pixel size (Å)	0.693	0.698	0.732	
Symmetry imposed	C1	C1	C1	
Initial particle images (no.)	8,932,246	4,358,411	4,006,243	
Final particle images (no.)	93,759	87,246	106,684	201,206
Map resolution (Å)	3.1	3.2	3.1	2.9
FSC threshold	0.143	0.143	0.143	0.143
**Refinement**				
Initial model used (PDB code)	RnPepT2 (7NQK)			
Model composition in the asymmetric unit				
Non-hydrogen atoms	4667	4710	4714	4714
Protein residues	590	595	595	595
Ligands	25	25	29	29
Average *B* factors (Å^2)				
Protein	34.55	77.51	41.84	49.03
Ligand	42.62	87.05	39.31	52.96
R.m.s. deviations				
Bond lengths (Å)	0.004	0.004	0.003	0.002
Bond angles (°)	0.49	0.62	0.57	0.57
Validation				
MolProbity score	1.91	1.90	1.92	1.93
Clashscore	7.15	7.09	5.72	4.86
Poor rotamers (%)	0.20	0.40	1.60	2.00

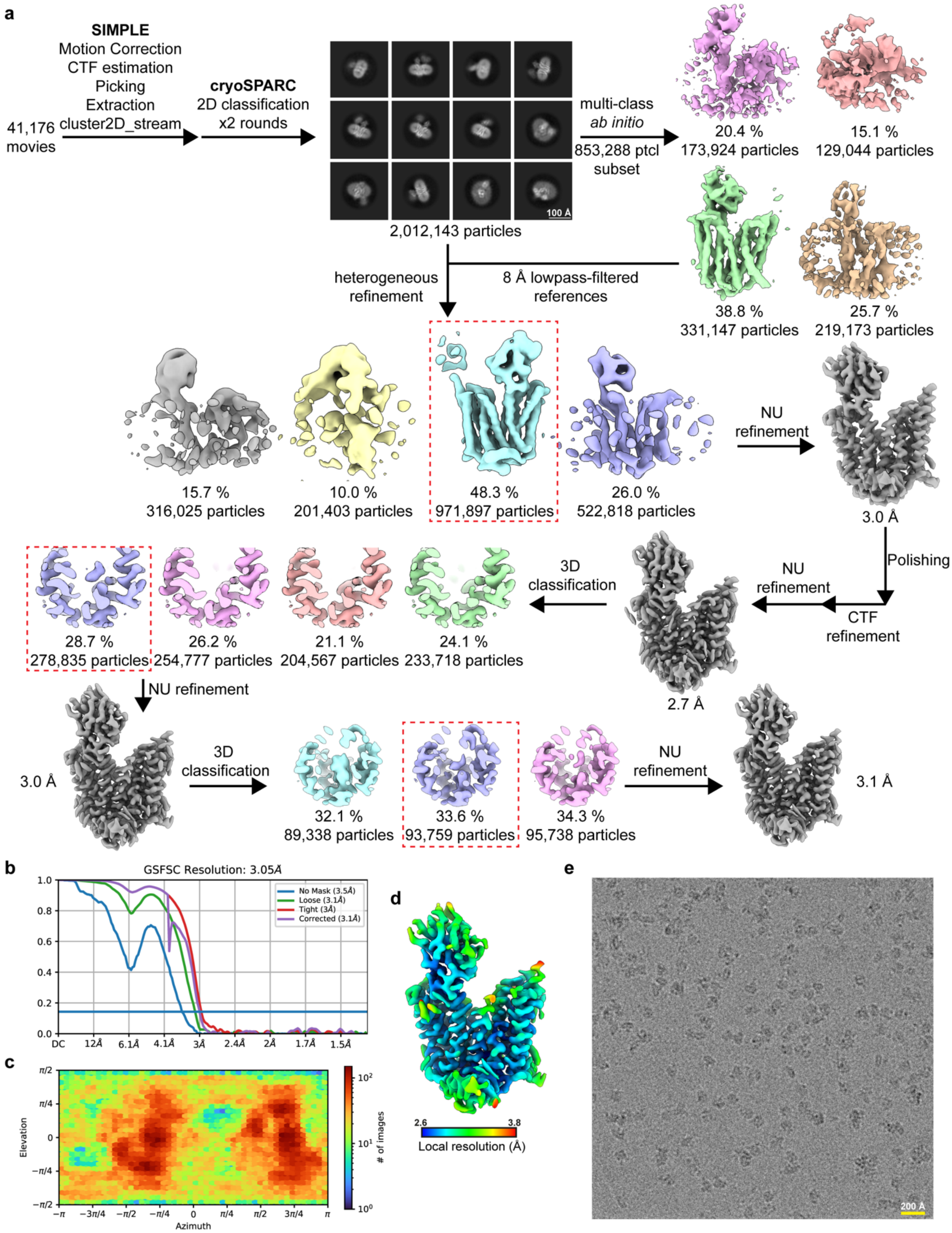

Supplementary Figure 1

Cryo-EM processing workflow of PepT2 with bound cefadroxil. **(a)** Image processing workflow. **(b)** Gold-standard Fourier Shell Correlation (FSC) curves for global resolution estimation. **(c)** Orientation distribution plot. **(d)** Local resolution estimate of the volume. **(e)** Representative micrograph.

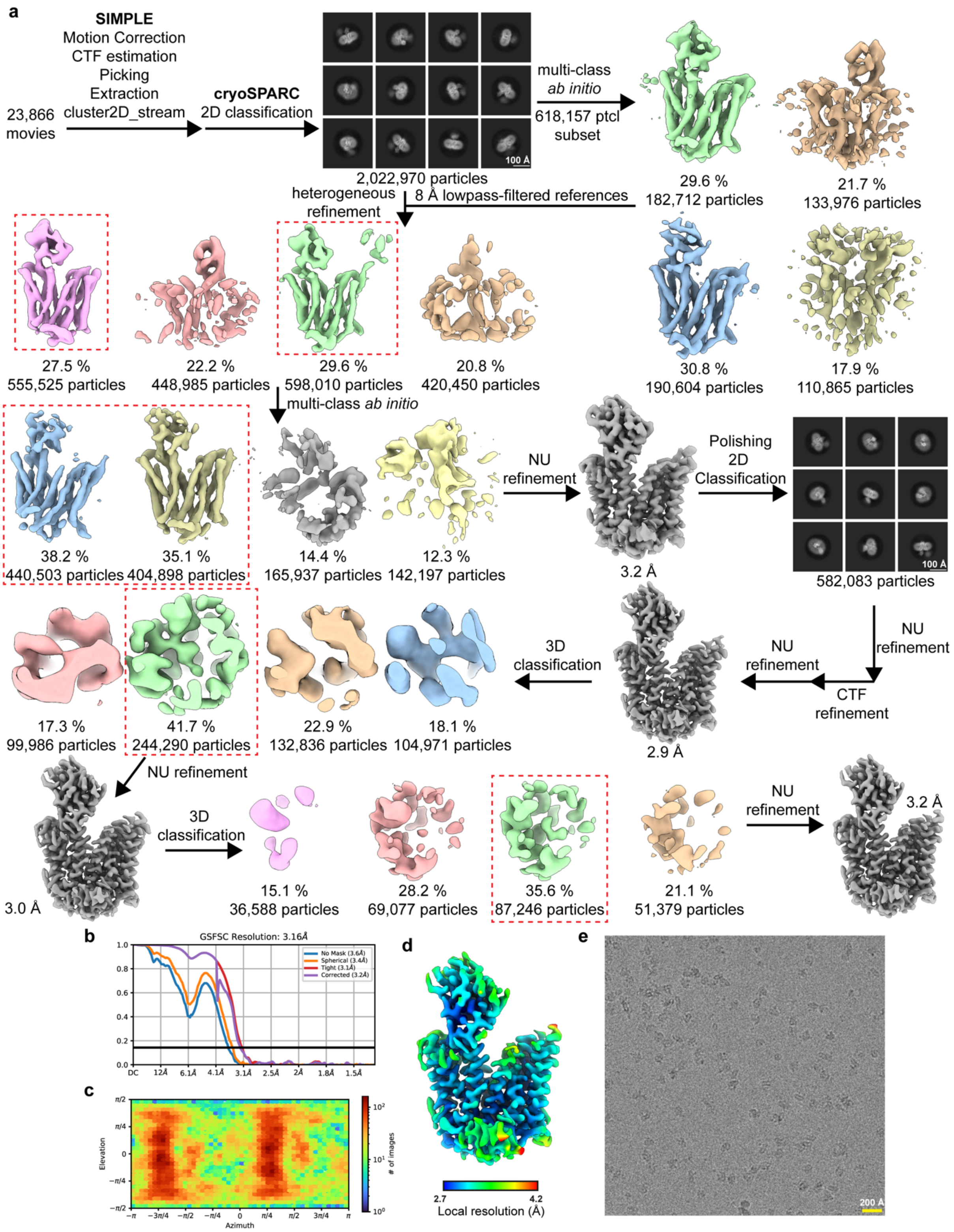

Supplementary Figure 2

Cryo-EM processing workflow of PepT2 with bound amoxicillin. (a) Image processing workflow. **(b)** Gold-standard Fourier Shell Correlation (FSC) curves for global resolution estimation. **(c)** Orientation distribution plot. **(d)** Local resolution estimate of the volume. **(e)** Representative micrograph.

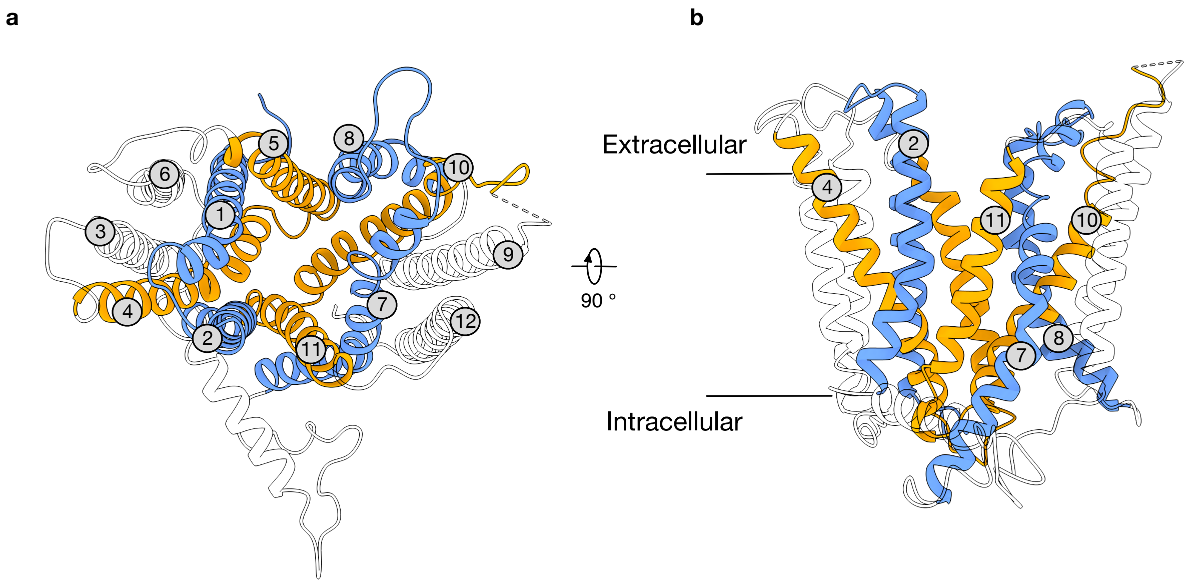

Supplementary Figure 3

Extracellular and intracellular gates in PepT2.

(a) The structure of RnPepT2 shows the location of the extracellular (blue) and intracellular (orange) gates. Transmembrane helices are labelled in grey circles. **(b)** View of the transporter in the plane of the membrane. The gating helices are labelled.

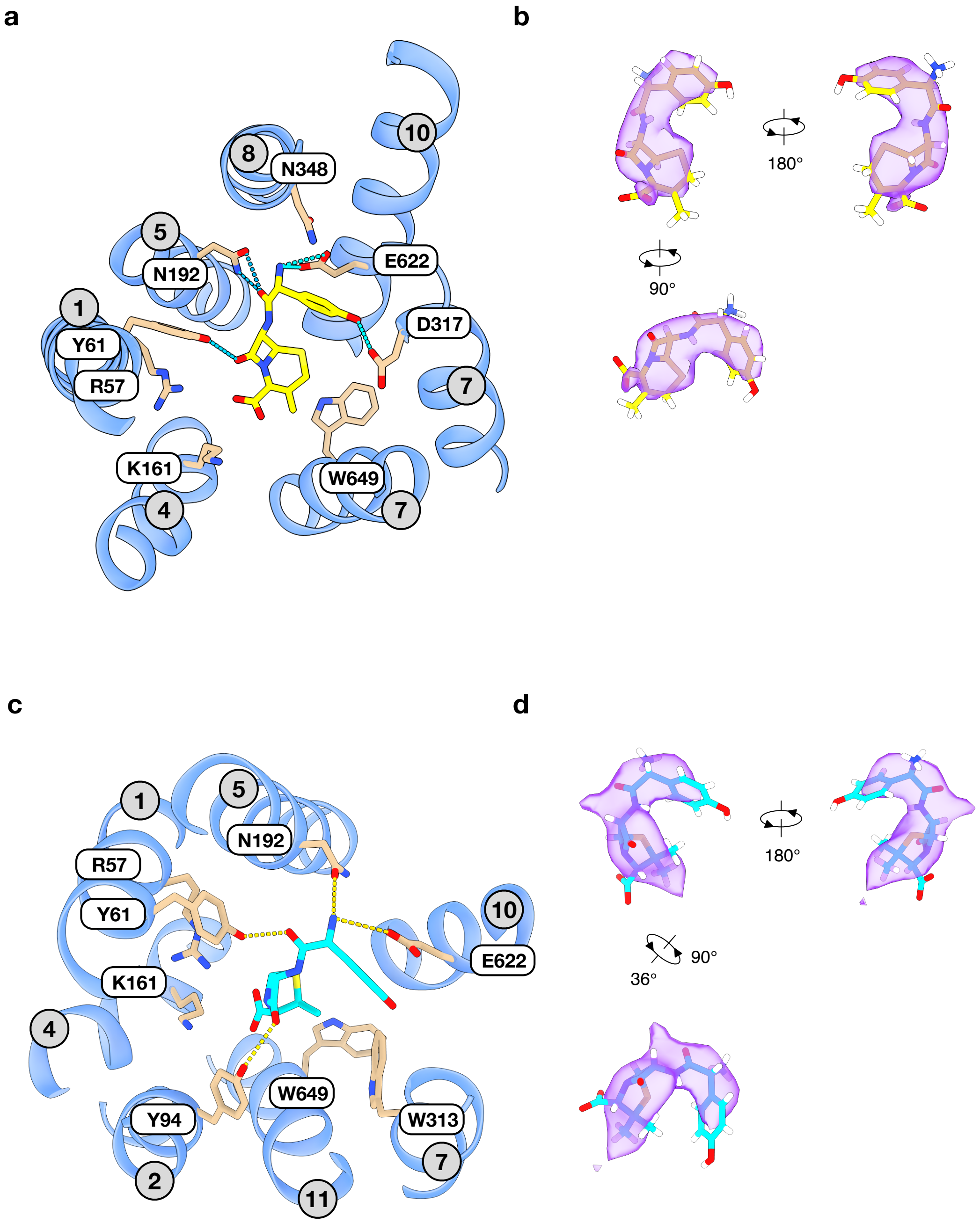

Supplementary Figure 4

Cryo-EM structure of antibiotic-PepT2 complexes. **(a)** The binding site of PepT2 shows the bound cefadroxil antibiotic (yellow sticks) with nearby and interacting side chains. Hydrogen bonds are denoted by dashed lines (cyan) and transmembrane helices labelled (grey circles). **(b)** Views of the cryo-EM density obtained for cefadroxil are shown, contoured at a threshold of 0.487. **(c)** The binding site of PepT2 shows the bound amoxicillin (cyan sticks). Hydrogen bonds are denoted by dashed lines (cyan) and transmembrane helices labelled (grey circles). **(d)** Views of the cryo-EM density obtained for amoxicillin are shown, contoured at a threshold of 0.442

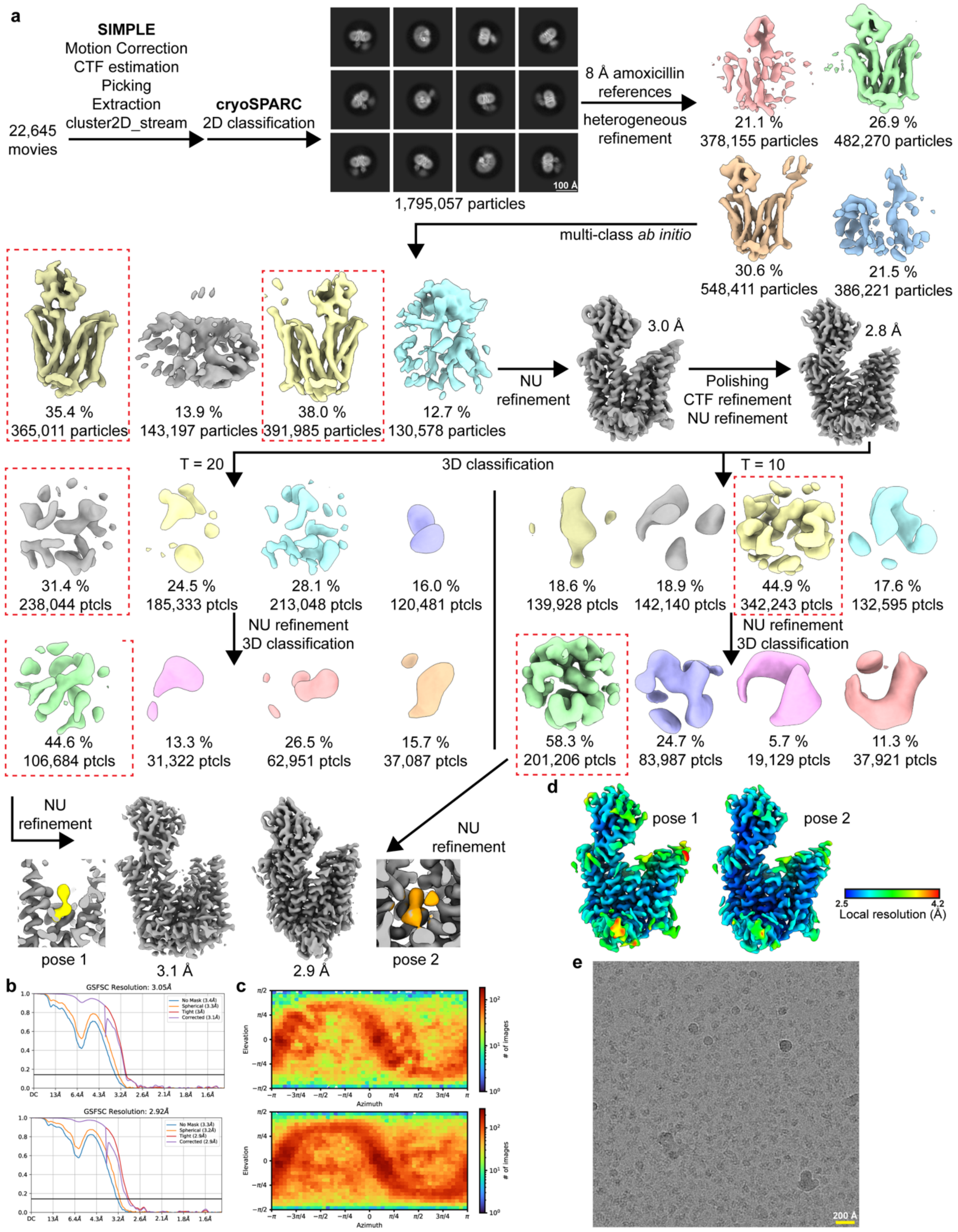

Supplementary Figure 5

Cryo-EM processing workflow of PepT2 with bound cloxacillin. **(a)** Image processing workflow. **(b)** Gold-standard Fourier Shell Correlation (FSC) curves used for global resolution estimation of pose 1 volume (top) and pose 2 volume (bottom). **(c)** Orientation distribution plots (pose 1, top; pose 2, bottom) **(d)** Local resolution estimate of the two volumes (pose 1; left, pose 2; right). **(e)** Representative micrograph

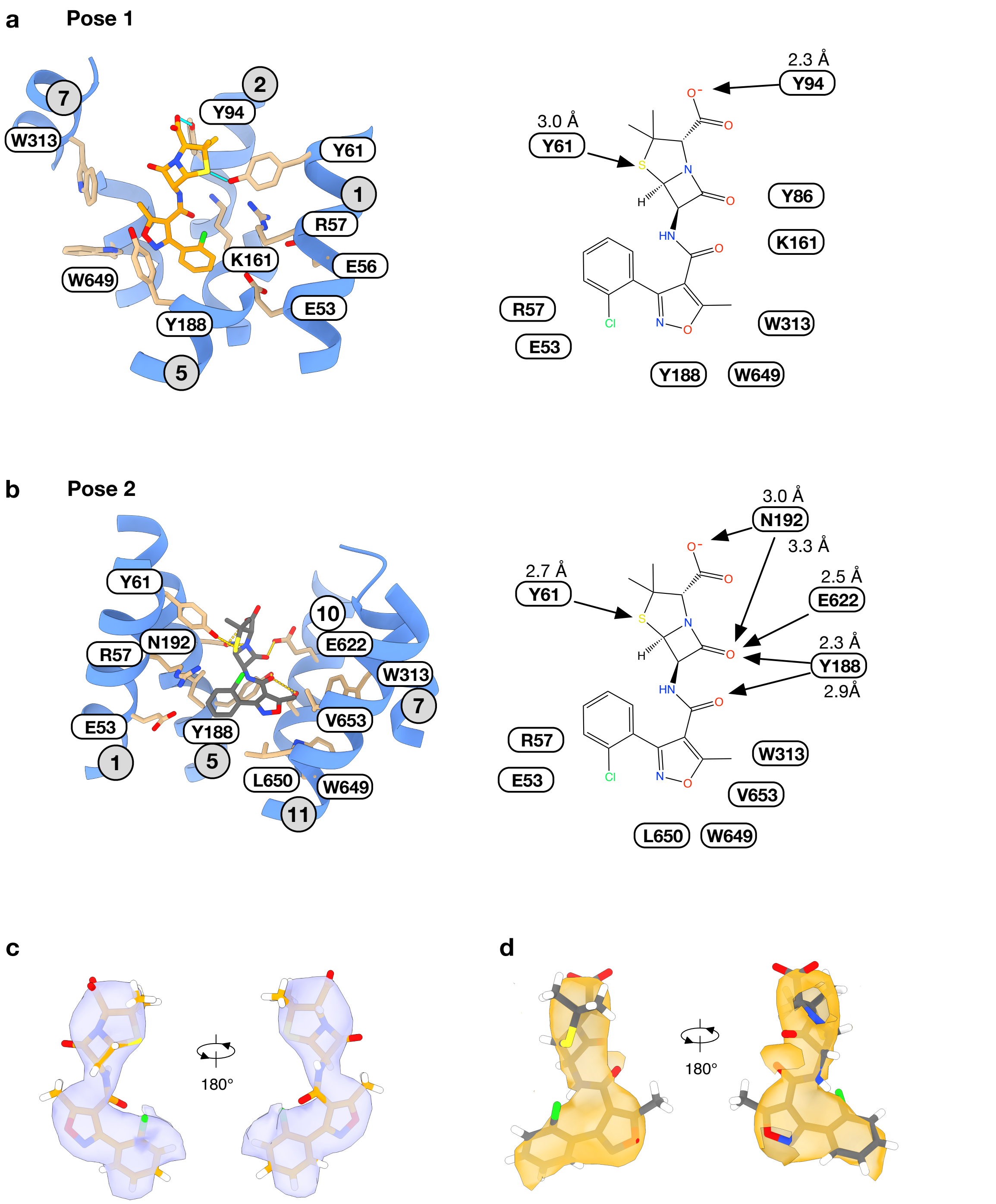

Supplementary Figure 6

Analysis of PepT2 cloxacillin interactions for poses 1-2. (a & b) Hydrogen bonds are shown as arrows denoting donor or acceptor atoms with distances (Å) indicated between heavy atoms. **(c)** Views of the cryo-EM density for pose 1, contoured at a threshold level ~ 0.22. **(d)** Views of the cryo-EM density for pose 2, contoured at a threshold level ~ 0.22. Poses 1 and 2 are however likely just two of several positions cloxacillin can adopt within the binding site.

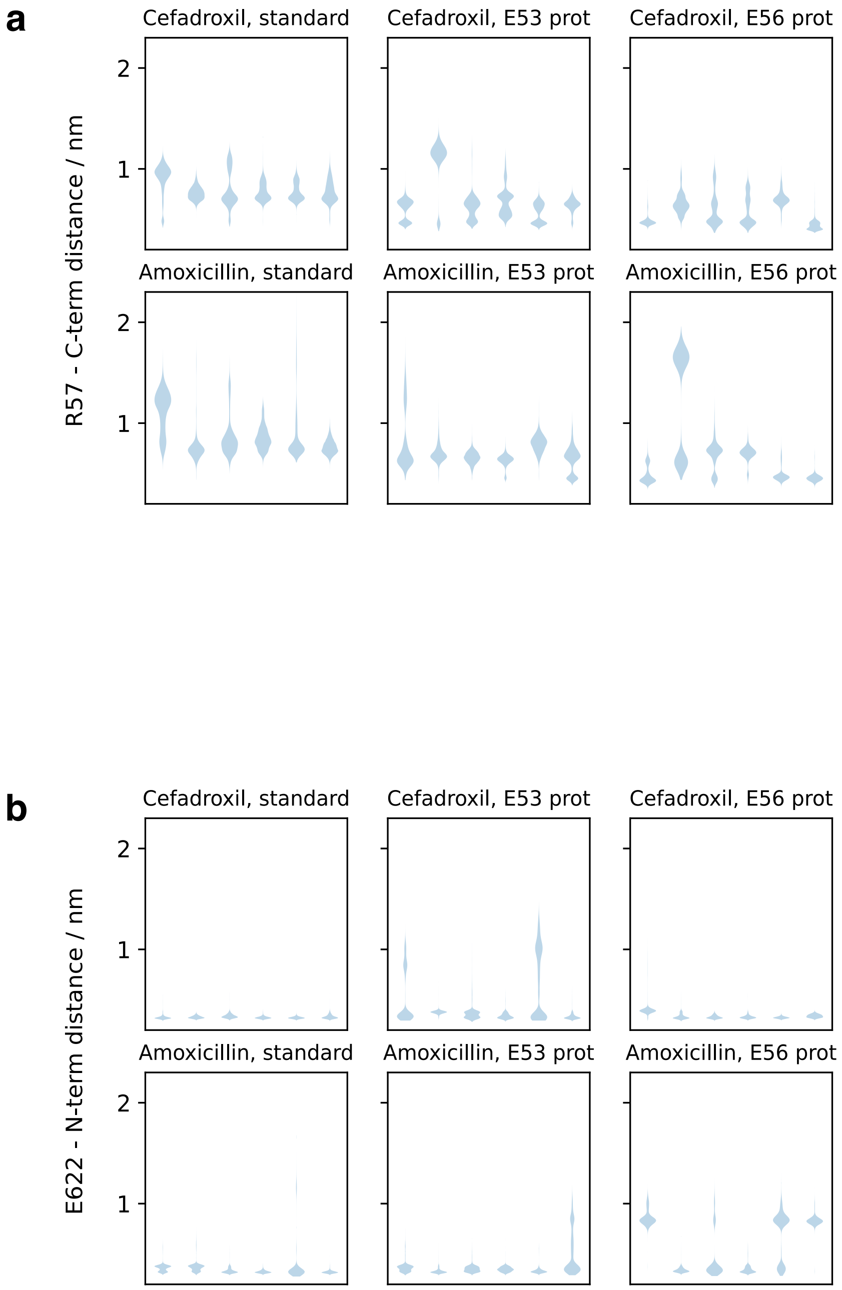

Supplementary Figure 7

Microsecond-long unbiased molecular dynamics (MD) simulations starting at the cefadroxil and amoxicillin cryo-EM models, using 6 replicates for each condition (standard protonation state, E53 protonated and E56 protonated). (a) Violin plots showing the individual replicate trajectory histograms for the data presented in figure 5A, using the drug carboxyl carbon – R57 (Cζ) distances and **(b)** the drug N-terminus (amino N) – E622 (Cδ) distances.

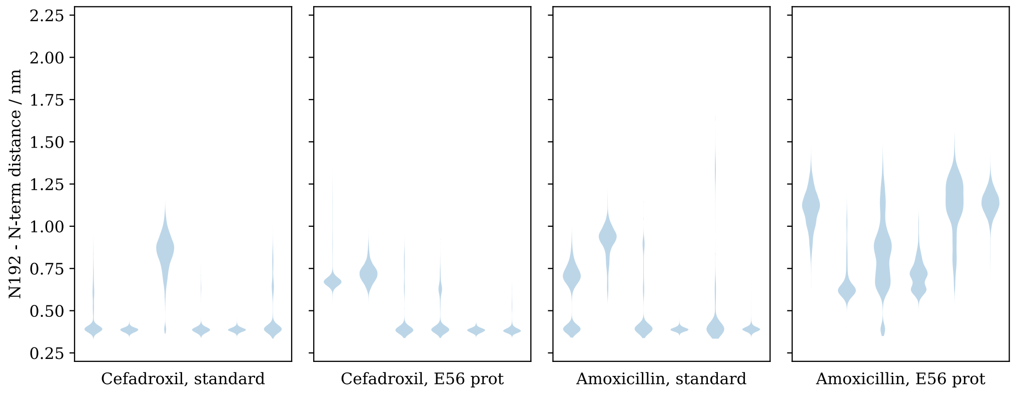

Supplementary Figure 8

Microsecond-long unbiased molecular dynamics (MD) simulations starting at the cefadroxil and amoxicillin cryo-EM models, using 6 replicates for the standard protonation state and E56 protonated conditions. Violin plots showing the individual replicate trajectory histograms for the drug N-terminus (amino N) – N192 (Cγ) distance.

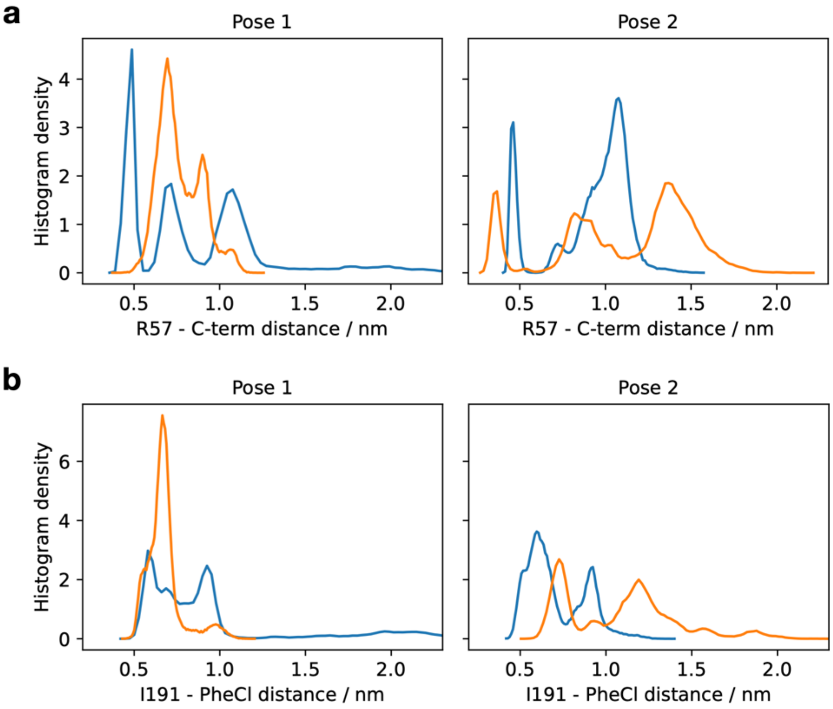

Supplementary Figure 9

Microsecond-long unbiased molecular dynamics (MD) simulations starting at the two cryo-EM models of cloxacillin, using 6 replicates for each model and condition (standard protonation state, E56 protonated). Histograms of the pooled trajectories of each pose / condition are shown for **(a)** the drug carboxyl carbon – R57 (Cζ) distance and **(b)** the drug chloride – I191 distance.

	**Counterflow activity relative to L-Ala-L-Ala**	**IC_{50} (μM) relative to L-Ala-L-Ala**
L-Ala-L-Ala	100	32
Amoxicillin	33	270
Cefadroxil	120	20
Cloxacillin	0	203
Moxalactam	0	500
Cefaclor	76	60
Ceftibuten	0	1200
Benzylpenicillin	0	1100
Ampicillin	13	750

Supplementary Table 2

Comparison of transport and inhibition properties of beta-lactam antibiotics with RnPepT2 using reconstituted liposome assay.
